# Nursing Workload in Systemic Anti‐Cancer Therapy Day Units: A Scoping Review and Gap Analysis

**DOI:** 10.1111/nhs.70330

**Published:** 2026-04-19

**Authors:** Zhuming Bao, Jenny Harris, Nianqi Cui, Verna Lavender, Anne Marie Rafferty, Jo Armes

**Affiliations:** ^1^ School of Health Sciences University of Surrey Surrey UK; ^2^ School of Nursing Kunming Medical University Kunming China; ^3^ Guy's Cancer Academy Guy's and St Thomas NHS Foundation Trust London UK; ^4^ King's College London London UK

**Keywords:** day units, nurse staffing levels, nurse workload, nursing workforce, SACT day units, scoping review, systemic anti‐cancer therapy

## Abstract

This scoping review aims to identify, summarize, and compare evidence from studies examining nursing workload in Systemic Anti‐Cancer Therapy (SACT) day units. The search included from January 2000 to 2025. Electronic databases, including MEDLINE, CINAHL, PsycINFO, EMBASE (Elsevier), ASSIA (ProQuest), Web of Science, Cochrane Library, and Global Index Medicus (WHO), were selected. A simplified search strategy was applied across platforms such as Open Gray, Google Scholar, Scopus, ProQuest Dissertations and Theses, and OAIster.20 studies were included in this review; among these, 16 studies were single centre. The studies were categorized into patient dependency workload (acuity tools, regimen‐based patient dependency, and patient‐based assessment) and nursing time workload. This review identified a significant gap in the literature, as the included studies largely failed to address non‐patient‐related care. It also highlighted that the complexity of SACT treatments influences nursing workload beyond patient numbers. This review underscores the distinct nature of nursing workload in SACT day units, the complexity and specialized nature of care required in SACT day units, and it highlights the need for refined workload measurement tools.

## Key Points

- This scoping review is the first known to provide a comprehensive overview of nursing workload in Systemic Anti‐Cancer Therapy (SACT) day units, categorizing existing measures and methodologies while highlighting significant gaps.
- This review identified significant gaps in the literature concerning nursing workload within SACT day units, notably the limited use of qualitative methodologies.
- This review highlighted that the identified workload measures failed to account for critical aspects of nursing work, particularly non‐patient‐related activities such as administrative, professional development, and coordination responsibility.

## 1 Introduction

The distinctive nature of nursing work significantly shapes both the quality and the level of care delivered to patients across various healthcare settings (Alghamdi 2016). Across all healthcare systems, nursing constitutes the largest healthcare profession and serves as the primary provider of patient care (Flaubert et al. 2021). Beyond administering treatments, nurses actively work to optimize patient outcomes by addressing patients' needs (Attenborough et al. 2019; Racy et al. 2021). However, persistent inadequate nurse staffing has been strongly associated with poorer patient outcomes (Greaves et al. 2018). For instance, a literature review identified that lower staffing levels correlate with higher rates of adverse events, increased mortality, and a greater incidence of healthcare‐associated infections (Carayon and Gurses, 2008). Insufficient staffing not only compromises patient care but also has a detrimental effect on nurses' well‐being, often resulting in burnout and driving some to leave the profession altogether (Moloney et al. 2018). Addressing these challenges requires the development and implementation of effective nurse staffing models that account for the inherent complexity and multifaceted nature of nursing care, a defining characteristic of the profession (Alghamdi 2016).

The concept of care complexity is widely used to describe situations that introduce uncertainty and unpredictability within healthcare systems (Reguera‐Carrasco and Barrientos‐Trigo 2024). Factors contributing to this complexity have been categorized into patient‐related, organizational, and operator‐related elements (Guarinoni et al. 2014, 2015). At the operator level, nurses' skills, professional experience, and workloads are critical determinants. Consequently, accurately measuring nursing workload is therefore an essential component of effective workforce planning.

Systemic Anti‐Cancer Therapy (SACT) refers to the administration of anti‐cancer treatments that act on the body, including chemotherapy, immunotherapy, targeted therapy, hormone therapy, and other systemic agents (Tuna et al. 2015). In the United Kingdom, alongside many countries internationally, increasing numbers of patients with cancer have further intensified the demands placed on nursing staff (Bao et al. 2024). Most patients with cancer are now treated in SACT day units (elsewhere known variously as Infusion Clinics, Oncology, or Chemotherapy Day Units), where nurses spend most of their time providing direct patient care. Moreover, advances in healthcare delivery have led to the predominance of nurse‐led SACT day units, presenting unique and complex challenges for nurses in these settings. Addressing the increasing demands of a growing patient with cancer population necessitates the development of reliable and valid methods for forecasting nursing staff requirements. While the scoping review conducted by Jessica and Gobnait (2023) identified tools for determining appropriate nurse staffing within Ambulatory Hematology Oncology Day Units (AHODUs), where SACT is administered, it did not provide a comprehensive description of nursing workload within this context. This gap underscores the need for further research to better understand the multifaceted nature of workload in this setting.

While various definitions of nursing workload exist and different methods have been developed to measure it, there remains a limited understanding of nursing workload specific to SACT day units. This review will address this gap by providing a comprehensive overview of the literature on nursing workload specific to the unique demands of SACT day units.

## 2 Aims

This scoping review aims to identify, summarize, and compare evidence from studies examining nursing workload in SACT day units.

### Objectives

- To identify and describe the measures used to assess nursing workload in SACT day units and examine how these measures have been operationalized.
- To explore and synthesize qualitative evidence focusing on nursing workload in SACT settings, identifying emergent themes
- To characterize nursing workload in SACT day units, providing an overview of its defining features and contributing factors.

## 3 Methods

### 3.1 Review Design

A scoping review was conducted in accordance with the JBI methodology for scoping reviews (Peters et al. 2020) and which adhered to the Preferred Reporting Items for Systematic Reviews and Meta‐Analyses (PRISMA) extension guidelines for Scoping Reviews (PRISMA‐ScR) (Tricco et al. 2018) (Data S1). The review question was “what evidence exists regarding nursing workload in systemic anti‐cancer therapy (SACT) day units, and how do findings across studies compare?” A systematic and rigorous approach was adopted to identify relevant studies that met the pre‐defined eligibility criteria. These criteria were established prior to the review process and were explicitly aligned with the research question, ensuring a minimum acceptable design level for inclusion in the analysis (open science framework (OSF), DOI 10.17605/OSF.IO/DCMV3; INPLASY202550032). Ethical approval is not required for this scoping review as the study involves the analysis of publicly available literature and does not include human participants, identifiable personal data, or direct interactions with individuals.

### 3.2 Search Methods

The search strategy was designed to identify a broad range of published and gray literature relevant to the aim of the review. A combination of electronic and manual search methods was employed to comprehensively capture literature pertinent to the research topic and the nursing field. Electronic databases, including MEDLINE, CINAHL, PsycINFO, EMBASE (Elsevier), ASSIA (ProQuest), Web of Science, Cochrane Library, and Global Index Medicus (WHO), were selected based on their relevance and coverage of healthcare and nursing research (Bramer et al. 2017). The specific search terms utilized, for instance, in CINAHL, are detailed in Data S2.

To identify gray literature and unpublished studies, a simplified search strategy was applied across platforms such as Open Gray, Google Scholar, Scopus, ProQuest Dissertations and Theses, and OAIster (Data S3). In addition, Google search engines were used with basic keywords to locate white papers and government documents. A manual search of bibliographies and reference lists of articles identified during the electronic search was also conducted to supplement the search strategy.

### 3.3 Eligibility Criteria

The inclusion criteria specified that studies must be published in English or translated into English and fall within the time frame of January 2000 to November 2025. The starting date of 2000 was deliberately chosen to align with the historical introduction of Ambulatory Cancer Care into the United Kingdom (UK) National Health Service (NHS) in 2004 (Finch et al. 2023). This timeframe ensures the review captures relevant literature leading up to and following the implementation of this significant development in cancer care delivery.

Eligible studies comprised original research employing either quantitative or qualitative methodologies. Exclusion criteria encompassed opinion articles, secondary research such as literature reviews, unpublished studies, single case studies, methodological papers, study protocols, animal studies, and conference abstracts. Nevertheless, the reference lists of excluded studies were examined to identify any potentially relevant sources.

#### 3.3.1 Participants

Studies were included if they focused on participants who were SACT nurses working in day unit settings. This encompassed registered nurses across a range of professional grades, irrespective of specific job titles, to ensure a comprehensive understanding of the nursing workforce in these contexts.

#### 3.3.2 Setting

The review specifically targeted SACT day unit settings, with a focus on oncology and/or hematology units. Studies related to only radiation oncology nursing, recognized as a distinct area of practice, were excluded (Blay et al. 2002). Similarly, studies conducted in alternative care settings, such as home‐based, community‐based, or mobile SACT care, were not included. The review exclusively included adult SACT settings, excluding studies centred on pediatric care. Studies that pertained to SACT day unit settings but did not explicitly address the delivery of SACT were also excluded.

#### 3.3.3 Concepts

The primary concept underpinning this review was nursing workload, defined as “the amount of time and care that a nurse can devote (directly and indirectly) to patients, the workplace and professional development” (Alghamdi 2016). The review considered all activities performed by SACT nurses within day units, encompassing both scheduled and unscheduled care. Tasks not undertaken by SACT day unit nurses were excluded from the scope of this review.

### 3.4 Selection and Data Extraction Process

Search results were imported into EndNote to remove duplicates, after which the remaining studies were transferred to Covidence for title, abstract, and full‐text screening by two independent reviewers (NC, ZB). Data extraction included key elements such as authorship and results using Covidence systematic review software. The quantitative evidence extraction table was developed in accordance with the PRISMA Extension guidelines for Scoping Reviews and piloted by the two reviewers (NC, ZB) using five sample studies. Feedback from the pilot phase was incorporated to refine the extraction template for improved clarity and usability. In contrast, the extraction table for qualitative evidence followed the same PRISMA guidelines but was not piloted due to the limited number of identified studies.

**FIGURE 1:**
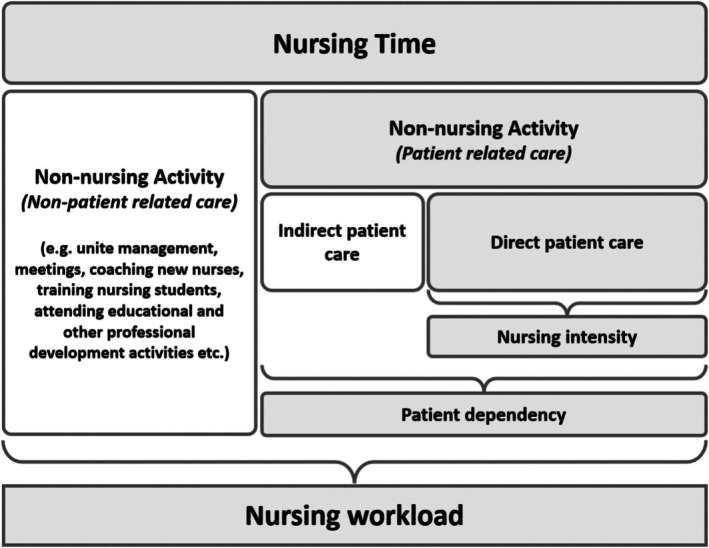
The distribution of nursing time and related concepts.(Fagerström and Rauhala, 2007).

The lead reviewer (ZB) conducted the remaining data extraction, which was subsequently verified by an independent second reviewer (NC) to ensure accuracy and reliability. The conceptual framework for understanding nursing workload among SACT day unit nurses in this study was informed by the Rainio, Fagerström, and Rauhala (RAFAELA) workload system (Fagerström and Rauhala, 2007). This system categorizes nursing workload into two primary domains: nursing activities, which include patient‐related care, and non‐nursing activities, which encompass tasks unrelated to direct patient care (Figure 1). The RAFAELA framework also facilitated the identification of key gaps in the literature concerning nursing workload in SACT day units, providing a structured lens through which these deficiencies were analyzed.

The finalized data extraction tables are presented in Appendix A and Appendix B. Additionally, Table 1, titled “Overview of Elements Included in Nursing Workload Measures Within SACT Day Units,” was collaboratively developed by the two reviewers (ZB, NC) in alignment with the RAFAELA workload system. This categorization framework divided workload elements into patient dependency (patient‐related care) and non‐nursing activities (non‐patient‐related care). The elements included in the table were systematically identified from all the studies reviewed, ensuring a comprehensive representation of workload components relevant to SACT day unit nursing.

**TABLE 1:** Overview of elements included in nursing workload within SACT day units.

				Blay et al. (2002)	Delaney et al. (2002)	Cusack et al. (2004)	Chabot and Fox (2005)	Moore and Hastings (2006)	DeLisle (2009)	Hawley and Carter (2009)	de Raad et al. (2010)	Green et al. (2012)	Kamimura et al. (2012)
Nursing time	Chair time			No	No	No	Yes	No	No	Yes	No	No	N/A
	Nursing time			No	No	Yes	Yes	Yes	Yes	No	Yes	Yes	N/A
	IV fluids (e.g., hydration)			No	No	No	No	Yes	Yes	No	No	Yes	N/A
	Drug infusion time			Yes	Yes	No	Yes	No	Yes	Yes	No	No	N/A
	Infection control			No	No	No	No	No	No	No	No	Yes	N/A
	Patient dependency	Direct care	Patient assessment	Yes	No	Yes	No	Yes	Yes	No	Yes	Yes	N/A
			Toxicity assessment	No	No	No	No	Yes	No	No	No	Yes	N/A
			Pre‐meds	No	No	No	No	Yes	No	Yes	No	Yes	N/A
			Venous access/care	Yes	No	Yes	No	Yes	Yes	Yes	Yes	Yes	N/A
			Patient bedside checks	Yes	No	No	No	No	No	No	No	No	N/A
			Continuous monitoring	No	No	No	No	Yes	No	No	Yes	No	N/A
			Medication education	No	No	No	No	No	No	No	No	No	N/A
			Side effects/reaction	No	No	No	Yes	Yes	No	Yes	Yes	Yes	N/A
			Phlebotomy	No	No	Yes	No	Yes	Yes	Yes	No	Yes	N/A
			Blood products	Yes	No	Yes	No	Yes	Yes	Yes	No	No	N/A
			Patient education	Yes	No	Yes	No	Yes	Yes	Yes	Yes	Yes	N/A
			Psychosocial support	No	No	Yes	Yes	Yes	No	No	No	No	N/A
			Symptom management	No	No	No	No	No	No	No	Yes	No	N/A
			Treatment related advice	No	No	No	No	No	No	No	No	No	N/A
			Provide general information	No	No	No	No	No	No	No	Yes	No	N/A
			Additional patient needs	Yes	No	No	No	Yes	No	Yes	Yes	Yes	N/A
			New patient (cycle 1)	No	No	No	Yes	No	Yes	No	Yes	Yes	N/A
		Indirect care	Coordination of care	Yes	No	Yes	No	Yes	Yes	Yes	No	Yes	N/A
			Drug checks	Yes	No	No	No	No	No	No	No	Yes	N/A
			Consent	No	No	No	No	No	No	No	No	Yes	N/A
			Records since last cycle	No	No	No	No	No	No	No	No	Yes	N/A
			Documentation	Yes	No	No	No	No	Yes	No	No	Yes	N/A
			Referrals	No	No	No	No	No	No	No	Yes	No	N/A
			Continuous care plan	Yes	No	No	No	No	No	Yes	Yes	Yes	N/A
			Telephone support/triage	Yes	No	Yes	No	No	No	No	No	No	N/A
	Non‐nursing activity (Non‐patient related)		Meetings	No	No	No	No	No	No	No	No	No	N/A
			Coaching new nurses	No	No	No	No	No	No	No	No	No	N/A
			Training nursing students	No	No	No	No	No	No	No	No	No	N/A
			Attending educational training	No	No	No	No	No	No	No	No	No	N/A
			Professional development	No	No	No	No	No	No	No	No	No	N/A

				de Souza et al. (2014)	Martin and Gaidzinski (2014)	Tuna et al. (2015)	Vortherms et al. (2015)	Baril et al. (2016)	Edwards (2017)	Santos and Gaidzinski (2019)	Cheevers et al. (2020)	Knox and Larmet (2021)	Bao et al. (2024)
Nursing time	Chair time			No	No	No	No	No	No	No	No	No	No
	Nursing time			Yes	No	Yes	No	Yes	No	Yes	No	Yes	Yes
	IV fluids (e.g., hydration)			No	Yes	Yes	No	No	Yes	No	No	Yes	Yes
	Drug infusion time			Yes	Yes	No	No	No	Yes	Yes	No	No	Yes
	Infection control			No	Yes	No	Yes	No	No	Yes	Yes	Yes	No
	Patient dependency	Direct care	Patient assessment	No	No	No	Yes	Yes	No	Yes	Yes	Yes	Yes
			Toxicity assessment	No	No	No	No	No	No	No	Yes	Yes	Yes
			Pre‐meds	No	No	No	No	No	No	No	No	Yes	Yes
			Venous access/care	Yes	Yes	Yes	Yes	Yes	Yes	Yes	Yes	Yes	Yes
			Patient bedside checks	No	No	No	No	Yes	No	Yes	No	No	Yes
			Continuous monitoring	Yes	No	No	No	Yes	No	Yes	Yes	No	Yes
			Medication education	No	Yes	No	No	No	No	No	Yes	No	Yes
			Side effects/reaction	No	No	Yes	No	No	No	No	Yes	Yes	Yes
			Phlebotomy	Yes	Yes	No	No	No	No	No	No	No	Yes
			Blood products	No	Yes	Yes	Yes	No	No	No	No	Yes	Yes
			Patient education	No	Yes	Yes	No	No	Yes	No	No	Yes	Yes
			Psychosocial support	No	No	No	No	No	No	No	No	No	Yes
			Symptom management	No	Yes	No	No	Yes	No	Yes	Yes	Yes	Yes
			Treatment related advice	No	Yes	No	No	Yes	No	Yes	No	No	Yes
			Provide general information	Yes	Yes	No	No	Yes	No	Yes	No	No	Yes
			Additional patient needs	Yes	Yes	No	Yes	Yes	No	Yes	No	Yes	Yes
			New patient (cycle 1)	No	No	No	No	No	No	No	No	Yes	Yes
		Indirect care	Coordination of care	Yes	Yes	Yes	Yes	Yes	No	Yes	Yes	Yes	Yes
			Drug checks	No	No	No	No	Yes	No	Yes	No	No	Yes
			Consent	No	No	No	No	No	No	No	No	No	Yes
			Records since last cycle	No	No	No	No	No	No	No	No	Yes	Yes
			Documentation	Yes	Yes	No	No	Yes	No	Yes	Yes	Yes	Yes
			Referrals	No	No	No	No	No	No	No	Yes	Yes	Yes
			Continuous care plan	Yes	No	No	No	No	No	No	Yes	Yes	Yes
			Telephone support/triage	Yes	Yes	No	No	Yes	No	Yes	Yes	No	Yes
	Non‐nursing activity (Non‐patient related)		Meetings	No	No	No	No	No	Yes	Yes	No	No	Yes
			Coaching new nurses	Yes	Yes	No	No	No	Yes	Yes	No	No	Yes
			Training nursing students	Yes	Yes	No	No	No	Yes	Yes	No	No	No
			Attending educational training	No	No	No	No	No	No	No	Yes	No	Yes
			Professional development	No	No	No	No	No	Yes	Yes	Yes	No	Yes

### 3.5 Data Analysis and Synthesis

The included studies were systematically summarized and organized into thematic categories guided by their relevance to the review's aim. The RAFAELA workload system was used as the analytical framework to structure the findings (Rauhala and Fagerström, 2007). Descriptive analysis was conducted to identify and highlight similarities and differences in the characteristics of the included studies.

To facilitate the clear presentation of key findings, tables were developed to chart and visualize the extracted data (Peters et al. 2020). These tables provided an organized overview, enabling a comprehensive mapping of the literature. As a scoping review, a formal quality appraisal of the included studies was not conducted because the primary purpose was to provide a descriptive mapping of the existing evidence and identify gaps rather than to evaluate the methodological quality of individual studies (Munn et al. 2018).

### 3.6 Ethical Considerations

This is a scoping review of previously published summary data; ethical approval for this study is not needed.

## 4 Results

### 4.1 Search Results

**FIGURE 2:**
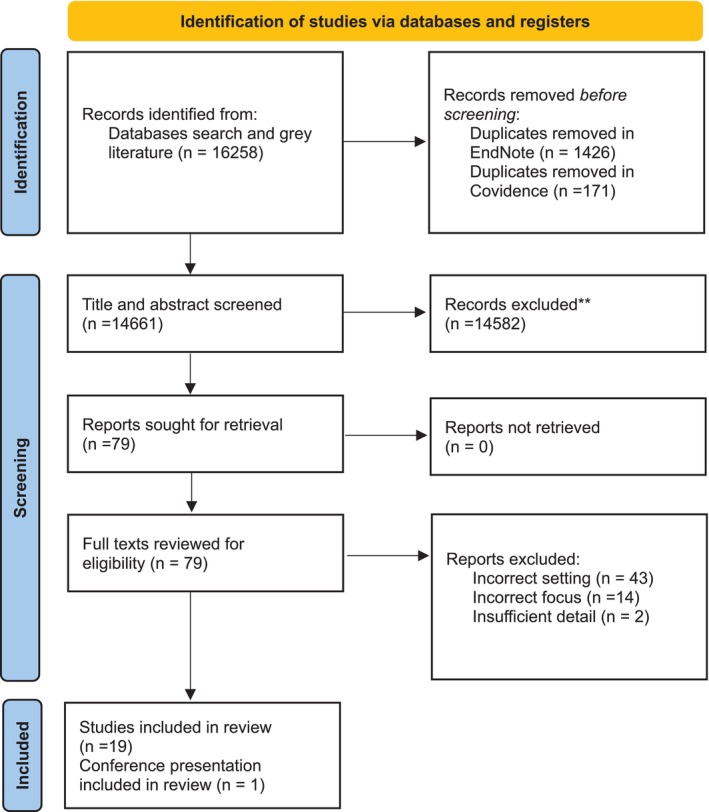
Study selection process for nursing workload in SACT day units.

A total of 16 258 records were identified through searches of electronic databases and gray literature sources. After removing 1597 duplicate records, 14 582 records were removed by title and abstract screening. The full text of 79 records was retrieved resulting in 20 studies (Appendixs A and B). The detailed screening and selection process is depicted in Figure 2.

### 4.2 Characteristics of the Included Studies

All studies were published between 2002 and 2024. The studies spanned six countries, with the largest representation from the United States (*n* = 8), followed by Australia (*n* = 3), Canada (*n* = 3), Brazil (*n* = 3), Turkey (*n* = 1), and the United Kingdom (*n* = 2). The settings examined across studies included chemotherapy outpatient units (*n* = 10), outpatient cancer centres (*n* = 5), and hematology‐oncology day care units (*n* = 5).

The registered nurse sample sizes included in the review ranged from 6 to 36 across multiple studies (Bao et al. 2024; Cheevers et al. 2020; de Souza et al. 2014; de Raad et al. 2010; Kamimura et al. 2012; Knox and Larmet 2021; Martin and Gaidzinski, 2014; Santos and Gaidzinski 2019). In contrast, studies conducted by Delaney et al. (2002) and Moore and Hastings (2006) included patient participants, with sample sizes ranging from 134 to 375. However, nine studies did not explicitly report the exact number of registered nurses (RNs) included. Instead, they used general terms such as “all the RNs included” (Blay et al. 2002; Chabot and Fox 2005; Cusack et al. 2004; DeLisle 2009; Edwards 2017; Hawley and Carter 2009; Moore and Hastings 2006; Tuna et al. 2015; Vortherms et al. 2015), which limits the ability to assess the precise sample representation. Additionally, Green et al. (2012) referred to “an expert panel” without specifying the exact number of participants, further highlighting inconsistencies in sample size reporting across studies.

Three studies were conducted across multiple centres (Bao et al. 2024; Kamimura et al. 2012; de Raad et al. 2010), while the remaining 16 studies were single‐centre investigations (Blay et al. 2002; Baril et al. 2016; Chabot and Fox 2005; Cheevers et al. 2020; Cusack et al. 2004; Delaney et al. 2002; DeLisle 2009; de Souza et al. 2014; Edwards 2017; Hawley and Carter 2009; Knox and Larmet 2021; Martin and Gaidzinski, 2014; Moore and Hastings 2006; Santos and Gaidzinski 2019; Tuna et al. 2015; Vortherms et al. 2015). Additionally, one study (Green et al. 2012) did not provide details on how the workload measure was applied in a setting.

Eight studies employed mixed methods approaches, integrating both qualitative and quantitative methodologies to assess nursing workload (Chabot and Fox 2005; de Raad et al. 2010; Moore and Hastings 2006; DeLisle 2009; Hawley and Carter 2009; Green et al. 2012; Cheevers et al. 2020; Knox and Larmet 2021). Additionally, seven studies relied exclusively on observational methodologies (Blay et al. 2002; Cusack et al. 2004; Delaney et al. 2002; de Souza et al. 2014; Baril et al. 2016; Santos and Gaidzinski 2019; Tuna et al. 2015). Only two studies adopted a qualitative research design, focusing on the experiences and perceptions of nurses regarding workload in SACT day units (Bao et al. 2024; Kamimura et al. 2012). Meanwhile, two studies utilized a cross‐sectional design (Martin and Gaidzinski, 2014; Vortherms et al. 2015), and one study implemented a pre‐post design to evaluate changes in workload over time (Edwards 2017).

Among these studies, nine incorporated time and motion methodologies to assess nursing workload by systematically tracking the time spent on various nursing activities (Blay et al. 2002; Delaney et al. 2002; Cusack et al. 2004; Hawley and Carter 2009; de Raad et al. 2010; de Souza et al. 2014; Tuna et al. 2015; Baril et al. 2016; Santos and Gaidzinski 2019).

### 4.3 Nursing Workload Measures in SACT Day Unit

The measures identified in this review from the 20 studies are summarized in Table 1. Common nursing activities identified across these measures include patient assessment, venous access and care, and phlebotomy, etc. Notably, the measures varied in their design and the specific elements they measured, reflecting the diversity of approaches used to assess nursing workload in SACT day units. To facilitate a clearer understanding, all measures were categorized and discussed within two distinct groups (Table 2):

- Patient Dependency Workload: employs a classification system to group patients according to their care needs, using a critical indicator tool to systematically assess and categorize the level of nursing demand (Alghamdi 2016). These indicators are typically plotted on a scale ranging from one to six categories, with each category representing a different level of patient dependency and corresponding nursing time requirements. This method is widely used to predict nursing workload, operating on the principle that patients with greater acuity or higher dependency levels require increased nursing resources (Knaus et al. 1981; Cusack et al. 2004). Twelve studies used patient dependency‐based methods to calculate nursing workload in SACT day units. These measures were categorized into three groups for comparison: acuity tools, regimen‐based patient dependency and patient‐based assessment.
- Nursing Time Workload: These measures focus on quantifying the actual tasks, activities, and interventions performed by nurses. Unlike dependency‐based methods, this approach focuses on the time and effort required to carry out nursing activities. They capture aspects of workload that may not be directly related to the patient's clinical condition but are nonetheless essential to care delivery.

**TABLE 2:** Measures based on patient dependency for workload.

Group	Overall description	Sub‐group	Sources
Patient dependency (*n* = 12)	Patient illness focused scores used for workload calculations (acuity‐quality methods).	Acuity tool (*n* = 8)	Cusack et al. (2004); Chabot and Fox (2005); Moore and Hastings (2006); DeLisle (2009); Hawley and Carter (2009); Tuna et al. (2015); Vortherms et al. (2015); Edwards (2017)
		Regimen‐based tool (*n* = 3)	Delaney et al. (2002); Green et al. (2012); Knox and Larmet (2021)
		Patient‐based assessment (*n* = 1)	de Raad et al. (2010)
Nursing time (*n* = 8)	Workload scores focus primarily on nursing interventions (task activity and professional judgment methods).	N/A	Blay et al. (2002); Kamimura et al. (2012); de Souza et al. (2014); Martin and Gaidzinski (2014); Baril et al. (2016); Santos and Gaidzinski (2019); Cheevers et al. (2020); Bao et al. (2024)

#### 4.3.1 Patient Dependency Workload

##### 4.3.1.1 Acuity Tools

Eight studies employed acuity tools that relied on critical indicators to define acuity levels. These levels were represented on scales (ranging from one to three or one to six categories), each representing the degree of a patient's demand on nursing time within a given period. Seven studies adopted a 3–6 level scale to reflect nursing workload. Of these, three provided recommended acuity levels per nurse per day (DeLisle 2009; EEdwards 2017; Hawley and Carter 2009).

The Magnuson model, introduced in 2004, significantly contributed to the development of a patient acuity system tailored to the complexities inherent in SACT day units (Cusack et al. 2004). It categorizes patient acuity into six levels based on the time required for both direct and indirect nursing care activities, with nurses' workloads determined by the cumulative acuity levels of their assigned patients. Three studies (Moore and Hastings 2006; Hawley and Carter 2009; Tuna et al. 2015) adapted this model for their own specific setting. For example, Moore and Hastings (2006) incorporated factors such as treatment duration, medication complexity, and supportive nursing activities into their tool. Three additional studies (Chabot and Fox 2005; DeLisle 2009; Edwards 2017) used similar acuity‐based systems.

In contrast to these approaches, Vortherms et al. (2015) introduced a points‐based system. Specific tasks, such as administering multiple drugs or new chemotherapy regimens, were assigned points (maximum five per patient). The average nursing workload ranged between 16 and 20 points per registered nurse.

##### 4.3.1.2 Regimen‐Based Patient Dependency

This category focused on workload calculations based on chemotherapy regimens. Delaney et al. (2002) developed the Chemotherapy Basic Treatment Equivalent (CBTE) model, assigning a baseline value of one to ‘Etoposide short’ (Etoposide phosphate administered as a short bolus), which had the shortest median treatment duration of thirty minutes. Other regimens were assigned relative weightings by dividing their median duration by 30 min (Basic Treatment Equivalent (BTE)).

Green et al. (2012) introduced a consensus‐based method accounting for the diversity of chemotherapy drugs and regimens. Their model incorporated 13 dimensions of nursing care and used a formula to calculate nursing workload time. The study also accounted for multitasking by adjusting for the variability in the drug reaction probabilities and dosing times.

Knox and Larmet (2021) adapted Green et al.'s (2012) methodology, organizing workload calculations into three appointment types: (1) new patients, (2) follow‐ups, and (3) active treatments. They further categorized new and follow‐up patients into low care needs and moderate care needs, recognizing that different patient groups require varying levels of nursing workload (Knox and Larmet 2021).

##### 4.3.1.3 Patient‐Based Assessment

This category centred on workload experienced in relation to individual patient care. De Raad et al. (2010) used surveys to identify key nursing tasks involved in administering chemotherapy such as patient education, assessment, administration, and communication. This provided views of the nursing workload specific to chemotherapy administration within SACT day units.

#### 4.3.2 Nursing Time Workload

Eight studies were identified that used nursing activities and tasks to represent the nursing workload within SACT day units. Two studies incorporated the concepts of direct and indirect care. Blay et al. (2002) evaluated nursing workload by categorizing tasks into direct care (*n* = 24) and indirect care (*n* = 26). Similarly, de Souza et al. (2014) investigated the distribution of time spent on direct and indirect care, further detailing time allocated to personal activities and associated tasks. Baril et al. (2016) adopted a different approach, classifying nursing activities into value‐added and non‐value‐added tasks, aiming to address the health needs of individuals or groups (Hobgood et al. 2005). Santos and Gaidzinski (2019) applied the Workload Indicators of Staffing Need (WISN) methodology, which included 35 nursing interventions and three additional activities: personal time, standby time and movement.

Two studies adapted existing instruments to align with the specific context of SACT day units. Martin and Gaidzinski (2014) validated an instrument to measure the time nursing staff spent on interventions and activities, selecting 26 items from the Nursing Interventions Classification (NIC) to provide a structured framework for workload measurement. Meanwhile, Cheevers et al. (2020) modified the Registered Nurse Forecast (RN4CAST) nurse survey aimed at evaluating nursing workload in SACT day units. However, this was a preliminary study conducted in a single cancer centre and was not used to examine associations between workload and the broader work environment. A distinctive feature of Cheevers et al.'s (2020) study was the inclusion of missed care as a component of workload assessment, linked to patient experience. This adaptation of the RN4CAST framework, originally developed by Sermeus et al. (2011), offers an approach to assessing the correlates and potential associations between workload, care quality, and occupational conditions within this specialized setting.

Two qualitative studies provided deeper insight into various aspects of nursing workload from the perspective of nurses, particularly in relation to the time required to deliver high‐quality care. Kamimura et al. (2012) conducted a qualitative study using two focus groups comprising 13 nurses, highlighting key challenges contributing to increased workload pressures. The study reported that temporary absences due to vacations, resignations, or sick leave disrupted staffing levels, leading to heightened workload demands. Additionally, uneven patient distribution was perceived as a major factor influencing workload intensity and creating challenges in maintaining high standards of care.

These findings were further supported by Bao et al. (2024), who explored the roles and responsibilities of nurses in SACT day units, identifying four distinct roles: the specialist role, leadership role, enabling work role, and supportive role. This study mapped out the distribution of nursing time and classified workload into patient‐related care and non‐patient‐related care. The specialist and supportive roles encompassed direct and indirect patient care, while the leadership and enabling work roles reflected non‐patient‐related care, such as professional development and care coordination. Furthermore, delays and missed care emerged as key indicators of nursing workload. Delays were found to interrupt nursing workflows, consequently increasing workload, while missed care signaled heightened work pressures, requiring nurses to prioritize essential tasks over others (Bao et al. 2024). This study also mapped out the typical patient workflow for an individual receiving care in a single SACT day unit, providing a structured overview of the sequence of nursing interventions and tasks involved in the treatment process (Bao et al. 2024).

### 4.4 Characterization of Nursing Workload

All studies found that nurses generally spent more time on indirect care than direct care. Blay et al. (2002) reported that 73% of nurses' time was allocated to indirect care, compared to 27% for direct care while excluding chemotherapy administration. Similarly, de Souza et al. (2014) found that indirect care accounted for 40% of total interventions and activities, while direct care comprised 34%, while associated work and personal activities accounted for 26%. De Raad et al. (2010) reported that patients received an average of 3.3 h of nursing staff time, divided into 1.7 h of direct contact and 1.6 h of non‐contact time. Baril et al. (2016) indicated that direct care represented 27% of nursing activities, while indirect care made up 65%, encompassing tasks such as clinical assessments, counseling and documentation. Moore and Hastings (2006) reported that time‐intensive indirect care interventions could account for as much as 0.8 Full‐Time Equivalent (FTE).

The most frequent or time‐consuming nursing activities varied across studies. Blay et al. (2002) identified medication checks, observations, and intubations as the most frequent direct care activities. de Souza et al. (2014) found that nurse consultations about treatments, chemotherapy administration, and pain management were the most time‐consuming direct care activities. Tuna et al. (2015) reported that nurses spent considerable time educating patients before treatment, establishing vascular access, and providing post‐treatment education. Indirect care activities such as documentation and care information change were identified as the largest contributors to care time in by both Blay et al. (2002) and de Souza et al. (2014).

Several studies reported the demands on nursing time. Santos and Gaidzinski (2019) observed that nurses spent 89% of their time on nursing interventions, 8% on personal activities, 3% on standby and 0.1% on movement. Baril et al. (2016) similarly reported that nurses were occupied 92% of their working time. However, some tasks did not require nursing expertise. For example, Baril et al. (2016) found that while 44% of indirect care tasks required nursing expertise, 21% could be delegated to ancillary staff. Cusack et al. (2004) also identified opportunities to reassign non‐specialist tasks, allowing nurses to focus on core responsibilities.

The complexity of SACT treatments was found to influence nursing workload beyond patient numbers, as demonstrated in three studies examining workload fluctuations within a single site across multiple days. Delaney et al. (2002) found that while the daily number of patients treated in chemotherapy outpatient centres remained relatively stable, treatment complexity varied significantly. There was wide variability in workloads, with nurses experiencing overburdened highly complex cases on some days and lighter workloads on others. Similarly, DeLisle (2009) identified a discrepancy between actual staffing levels and patient complexity, suggesting an acuity level of 20 points per nurse per day would ensure optimal workload distribution. Furthermore, Cheevers et al. (2020) highlighted the consequences of high workload pressures, identifying missed nursing care as a critical issue. The study reported that 54% of SACT nurses at a single centre had failed to assess toxicities before chemotherapy at least once a month. Other missed care elements included verbal patient communication, monitoring comorbidities, and toxicity assessments, reflecting the increased care complexity and unmet patient needs resulting from workload strain. These findings were further supported by Bao et al. (2024), which revealed that nurses devoted considerable time to keeping their clinical knowledge up to date with the rapid advances in SACT treatments. This necessity for continuous learning adds an additional layer of indirect workload, further emphasizing the multifaceted nature of nursing responsibilities within SACT day units.

**FIGURE 3:**
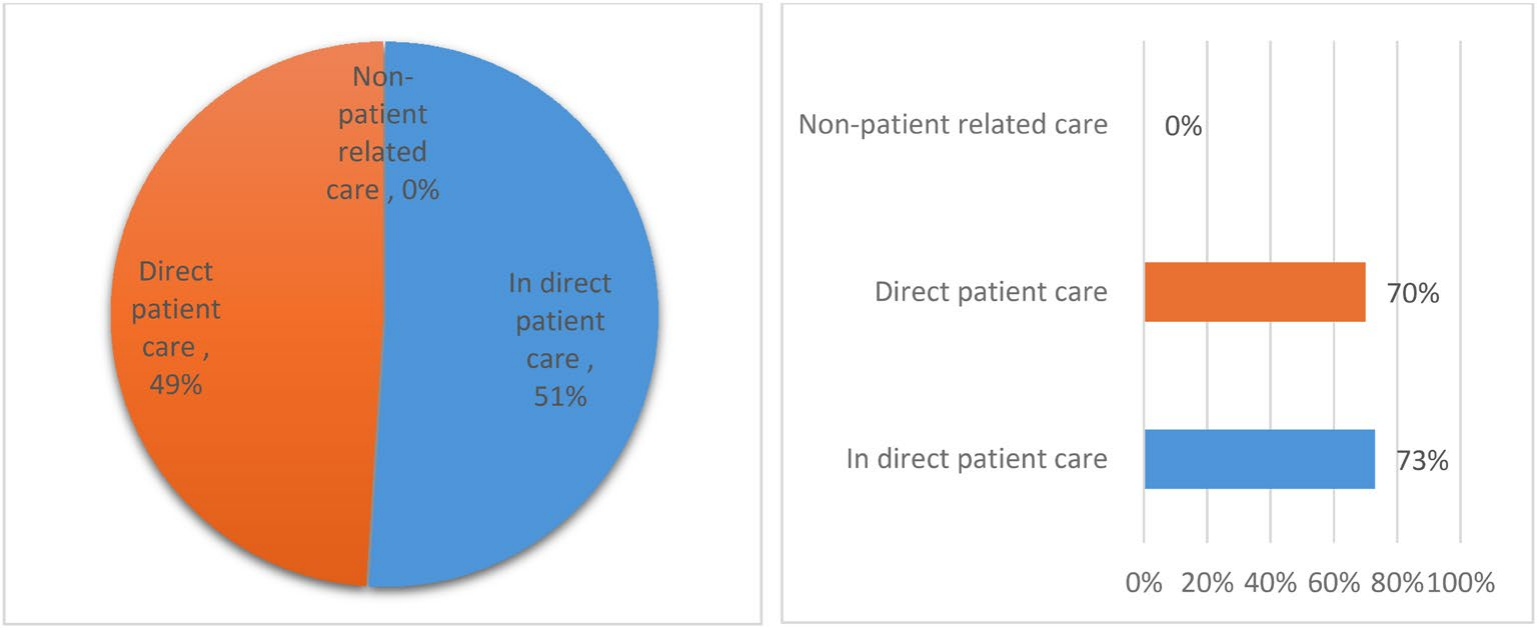
Coverage of nursing workload elements.

This review identified a significant gap in the literature, as the included studies largely failed to address non‐patient‐related care. The majority of studies focused primarily on patient dependency‐based workload, emphasizing nursing activities driven by patient care needs while overlooking the essential but less visible tasks required to maintain the functioning of SACT day units. These behind‐the‐scenes activities, which contribute to overall nursing workload, were largely absent from existing workload assessments. While 51% of studies examined indirect patient care, direct patient care was covered in 49% of studies (Figure 3). However, only six studies (Bao et al. 2024; Cheevers et al. 2020; de Souza et al. 2014; Santos and Gaidzinski 2019; Edwards 2017; Martin and Gaidzinski, 2014) mentioned one or a few elements of non‐patient‐related care, and even in these cases, the percentage of coverage remained extremely low. Furthermore, when assessing the identified workload elements across all included studies, 73% of indirect patient care activities were covered, compared to 70% of direct patient care activities.

## 5 Discussion

This scoping review is the first known to provide a comprehensive overview of nursing workload in SACT day units, categorizing existing measures and methodologies while highlighting significant gaps. Patient dependency measures were the most frequently used approach, particularly acuity‐based tools, reflecting a focus on classifying patient needs and direct care tasks (Hughes and Cutting 1999). However, this reliance on patient dependency alone fails to capture the broader spectrum of nursing responsibilities (Alghamdi 2016; Morris et al. 2007), particularly indirect care and non‐patient‐related activities (Alghamdi 2016; Begley et al. 2004). This limitation underscores the need for more comprehensive workload measures that reflect the full scope of nursing practice in SACT day units.

Observational studies, including time and motion methodologies, offered valuable insights into nursing time allocation but many were resource‐intensive and limited in scope (Lopetegui et al. 2014). These studies predominantly focused on task‐based, physical aspects of care while neglecting the relational, social, and psychological dimensions of nursing work (Lopetegui et al. 2014). Furthermore, many observational studies failed to account for the sequential or concurrent nature of nursing interventions, overlooking the complexity of workload distribution. The diversity of approaches identified in this review underscores the argument that no single measure that is universally applicable to comprehensively assess SACT workload (Griffiths et al. 2020), methods are required to unpick workload and its consequences in this complex setting.

The review identified significant gaps in the literature concerning nursing workload within SACT day units, notably the limited use of qualitative methodologies, with only two studies identified. These qualitative studies (Bao et al. 2024; Kamimura et al. 2012) provided essential insights into the lived experiences of frontline nurses, offering a nuanced understanding of workload complexities that quantitative approaches often fail to capture. Specifically, they illuminated contextual factors such as organizational dynamics, patient unpredictability, and the interdependence between care demands and resource constraints. This perspective is critical in highlighting challenges unique to SACT day units, including the management of delays, last‐minute schedule changes, and the necessity for real‐time care adjustments. Such insights underscore the relational and systemic dimensions of workload, which are inadequately addressed by task‐based or dependency‐focused metrics.

The review identified an important gap in the literature, the lack of attention to the unique workload challenges stemming from uncertainties in SACT day units. Unlike other healthcare settings, although patients rarely miss appointment, SACT nurses frequently manage patients who are unfit for treatment upon arrival, requiring additional care or rescheduling. Delays were identified as a significant source of uncertainty in SACT day units, exerting a substantial impact on nursing workflow (Bao et al. 2024). This dynamic increased workload but is rarely accounted for in these studies (Rodríguez‐Rey et al. 2020). Similarly, the predominance of indirect nursing care in SACT day units contrasts starkly with other settings, such as inpatient wards where, for example, Al‐Moteri et al. (2023) found that nurses spent 69% of their time on direct patient care during an eight‐hour morning shift and only 12% on indirect care. Likewise, Kakushi and Evora (2014) reported that intensive care unit nurses provided 27.4 h of direct care compared to 2.1 h of indirect care per patient per day. These findings emphasize the need to design workload measures tailored to the unique demands of SACT day units.

Moreover, many of the identified workload measures failed to account for critical aspects of nursing work, particularly non‐patient‐related activities such as administrative, professional development, and coordination responsibilities. This omission of non‐patient‐related activities highlights the need for future workload assessment tools to adopt a comprehensive approach that captures the full spectrum of nursing responsibilities, ensuring that both patient‐related and non‐patient‐related care are adequately represented. Cheevers et al. (2020) underscored the significance of addressing missed nursing care, a phenomenon often associated with excessive workloads (Blackman et al. 2017; Duffy et al. 2018). Missed nursing care such as essential tasks such as verbal communication with patients, monitoring comorbidities, and assessing toxicities are likely to negatively impact patient experience and outcomes but are yet to be comprehensively explored in the SACT unit context. Future studies of workload should incorporate these dimensions to provide a holistic understanding of nursing workloads.

A shift in the focus of workload measure development was observed with more recent studies emphasizing nursing time and contributions rather than solely on patient dependency. This evolution reflects growing recognition of the important, often unseen, contributions nurses make to achieving better patient outcomes and delivery of high‐quality care. Additionally, approaches such as linking workload to patient experiences (Cheevers et al. 2020) may offer promising avenues for understanding the broader implications of workload management.

## 6 Limitations

This review was limited to studies published in English and indexed in the selected databases between 2000 and 2025, which may have constrained the scope of nursing workload measures evaluated. A limitation of this study is that the included articles primarily focus on the delivery of care in adult anti‐cancer clinics. As such, the findings may not be generalisable to pediatric oncology settings or other sub‐specialty oncology clinics, such as survivorship clinics. Additionally, the reporting within several studies lacked sufficient detail regarding sample characteristics and the specific elements encompassed within their nursing workload measures. This limitation restricted the ability to conduct a comprehensive evaluation and comparison of their methodologies. The inclusion of studies from several countries introduces variability, making it challenging to generalize the findings across diverse healthcare systems. Moreover, the predominance of single‐centre studies further constrains the applicability of the results to broader contexts.

## 7 Conclusion

This scoping review identified the diverse and evolving nature of workload measurement approaches in SACT day units, highlighting methodological gaps and key areas for improvement. The findings underscore the distinct nature of nursing workload in SACT day units, the complexity and specialized nature of care required in SACT day units. It highlights the need for refined workload measurement tools that integrate patient‐related and non‐patient‐related activities, account for treatment complexity, and incorporate qualitative insights to support workforce planning and ensure sustainable nursing workload management in SACT day units. By refining and standardizing nursing workload measures, future research can provide a stronger evidence base for workload management strategies. Such advancements are important for supporting nurses in SACT day units, improving patient outcomes, and enhancing the sustainability of high‐quality cancer care delivery.

## 8 Relevance for Clinical Practice

The findings of this study highlight the need for the development of comprehensive workload measurement tools tailored to the context of SACT day units. Such tools should systematically account for both patient‐related and non‐patient‐related activities, reflect the complexity and unpredictability of SACT regimens, and integrate qualitative insights from nursing practice. Incorporating these dimensions will not only support more accurate and responsive workforce planning but also provide an evidence base to inform policy decisions regarding safe staffing and resource allocation in oncology services. Strengthening workload measurement in this way will contribute to sustainable workload management and ultimately enhance the delivery of safe, timely, and effective patient care within SACT day units.

## Author Contributions

**Zhuming Bao:** contributed to the design of the study, conceptualized the study, performed database searches, study selection and data extraction, analyzed and interpreted the data, and drafted the manuscript. **Jenny Harris:** contributed to the design of the study, contributed to the interpretation of the data, and revised the manuscript. **Nianqi Cui:** performed database searches, study selection and data extraction. **Verna Lavender:** supervised the study, guided the research methodology, critically reviewed the manuscript. **Anne Marie Rafferty:** supervised the study, guided the research methodology, critically reviewed the manuscript. **Jo Armes:** contributed to the design of the study, contributed to the interpretation of the data, and revised the manuscript. All authors approved the submitted version and agreed to take personal responsibility for their own contributions and for the accuracy and integrity of the work.

## Funding

This research is funded by the University of Surrey as a PhD studentship awarded to ZB. JA receives funding from the National Institute for Health and Care Research (NIHR) Applied Research Collaboration Kent, Surrey, Sussex. The views expressed are those of the author(s) and not necessarily those of the NHS, the NIHR, or the Department of Health and Social Care.

## Conflicts of Interest

The authors declare no conflicts of interest.

## Supporting information


**Data S1:** PRISMA‐ScR‐fillable‐checklist.

**Data S2:** Search terms for electronic databases.

**Data S3:** Simplified search term.

## Data Availability

The data that supports the findings of this study are available in the Supporting Information of this article.

## Appendix A Data Extraction Table for Quantitative Study.

**Table:**

Author/Date country	Study aims	Design	Nursing workload measures				Sample characteristics		Findings
			Name	Measure's focus	No. of items; scoring	Measure develop process	Sample/size	Setting	
Blay et al. (2002) (Australia)	To evaluate the roles and workload of the nurses in Outpatient Cancer Care Centre.	An observational study Data collection: timing nurse activity for 3 months. Self‐reported data (the frequency of tasks) was measured on a chart for 2 weeks. Single centre	N/A	To evaluate the roles and workload of the nurses in Outpatient Cancer Care Centre.	Direct care tasks *n* = 24, indirect care tasks *n* = 26	Brainstorming the nursing tasks Categorization of direct and indirect tasks Identified and categorized chemotherapy administrations by duration of administration.	Registered Nurses (*n* = unknown)	Hematology Oncology Day Care unit (HODC)	The time chemotherapy nurses spent: 72.5% on indirect care whereas 27.4% on direct care excluding chemotherapy administration. Administrative duties constitute a large proportion of the HODC nurses' time. Interpretation: Nurses in SACT day units spend huge amount of time on indirect care tasks, highlighting inefficiencies in workload distribution.
Delaney et al. (2002) (Australia)	To study the factors that impact on treatment episode duration for outpatient chemotherapy treatment delivery.	An observational study Data collection: (1) Stopwatch‐timed randomly selected patient episodes over a four‐week period. (2) Patient related data were collected. Single centre	Chemotherapy Basic Treatment Equivalent (CBTE) model	To assess the impact of various factors on outpatient chemotherapy treatment duration.	CBTE of 1 equal 30 min. All other chemotherapy regimens then are given a relative complexity ranking which is a multiple of 30 min.	N/A	Patient under chemotherapy (*n* = 134) Occasions of delivered service (*n* = 266)	Chemotherapy Day Centre	The complexity of treatment varies considerably compared to patient number. The nurses are perhaps overworked with complex treatments and less stressful when the complexity ranking per patient is low. The treatment duration was impacted most by the type of infusion and the chemotherapy regimen that has been prescribed. Interpretation: The complexity of SACT regimens significantly impacts nursing workload, emphasizing the need for tailored workload measures.
Cusack et al. (2004) USA	To develop a patient intensity system that reflected both the severity of patient illness/need and the complexity of service required.	A cross‐sectional observational study Data collection: (1) Five‐point acuity level tool; (2) An instruction sheet; 3); A staff satisfaction survey. Single centre	Ambulatory Intensity System (AIS), Magnuson Model	To quantify the nursing care requirements and to improve patient care delivery.	Final: Six‐point acuity level (1–6)	Nursing leadership chose the prototype system to adapt for the AIS. Objective retrospective quantification of time spent with patients. Five‐point acuity level tool was developed	Registered nurses (*n* = unknown)	Outpatient cancer centre (OCC)	The tool identified many tasks for which nursing had assumed responsibility. However, these tasks can be delegated to other ancillary staff, thereby affording the nurses more time for performing appropriate nursing activities. Interpretation: Delegating tasks previously managed by nurses in SACT settings can enhance nursing efficiency and focus on patient care.
Chabot and Fox (2005) USA	To estimate nursing time based on chemo complexity, balances total acuity per nurse in daily schedule.	A mixed methods study Data collection: used the Patient classification system (PCS) for 3 years Single centre	Patient classification system (PCS)	To provide specific treatments, special observations related to administration, and rate titration.	Three‐point acuity level (1–3) 30‐min acuity level, 60‐min acuity level, 90‐min acuity level.	Tool development: nurses' input, rely on objective retrospective quantification of time spent with patients.	Registered nurses (*n* = unknown)	Rower Hematology Oncology Medical Group outpatient infusion centre	The PCS reflects patient care needs and nursing care time necessary to meet such needs. The system matched nurses' scheduled hours with patient care time requirements. Interpretation: this system ignored the complexity of nursing workload.
Moore and Hastings (2006) USA	To establish a methodology for developing a user‐friendly, environmentally specific nursing intensity measurement tool.	A mixed methods study Data collection: daily entries of intensity ratings into the hospital's database for 4 months. Single centre	Hematology/Oncology Day Hospital Profile	To identify unique nursing intensity profiles.	Six‐point acuity level (1–6)	A team of stakeholders adopted the prototype AIS template (Cusack et al. 2004).	Registered nurses (*n* = unknown) Patients (*n* = 375)	Hematology oncology day hospital	Content validity index (CVI) was 0.87, indicating that descriptors were assigned to appropriate levels. Time intensive indirect care interventions represented as much as 0.8 Full Time Equivalent (FTE). Interpretation: Indirect care interventions consume a significant portion of nursing time in SACT units, emphasizing the need for efficiency improvements.
DeLisle (2009) USA	To describe the development of an acuity tool for staffing based on the complexity of patient treatments.	A mixed methods study Data collection: the revised acuity tool was implemented in May 2007 Single centre	Acuity Scale for oncology outpatient infusion room	To represent the various tasks, the tool was divided into three categories (the time required for that level, nonchemotherapy‐related activities, chemotherapy and treatment regimens).	Five‐point acuity level (1–5) A total acuity level of 20 points per nurse per day was suggested.	A tool was developed by the nursing team in the unit and tested for two months. After piloting: tool was revised by the team based on the nurse discussion	Registered nurses (*n* = unknown)	Ambulatory oncology infusion room with 11 clinics	The revised acuity tool was implemented and has proven to be useful in staffing the clinics. Actual staffing was not found to be an accurate representation of patient complexity when compared to proposed full‐time equivalent staffing. Interpretation: The revised acuity tool effectively supports SACT clinic staffing but highlights discrepancies between actual staffing and patient complexity.
Hawley and Carter (2009) USA	To enhance the patient experience in the infusion centre and to create a balanced workload for the Cancer Centre's infusion nurses.	A mixed methods study Single centre	The Cancer Centre's Medical Oncology Acuity of Care Rating System Adapted from Cusack et al. (2004), Moore and Hastings (2006)	To create a balanced workload for infusion nurses.	Five‐point acuity level (1–5) When the acuity level goes higher than 22, ideally another nurse is needed.	Staff logged all patients' movements for 2 weeks A practice council was established to adapt the existing tool. A “Treatment Length Template” was added after piloting.	Registered nurses (*n* = unknown)	Cancer infusion centre, 24 chairs	This number (22) translates to 6 to 8 patients per infusion nurse per day. Interpretation: this is a relatively small unit therefore the schedule system and the patient number consider small and probably easier to manage.
de Raad et al. (2010) Australia	To examine the nursing workload of administering alternative chemotherapy protocols as a driver of costs.	A mixed methods study Multi‐centres	Description of Nursing Activities within a chemotherapy Protocol	To capture most nursing activities associated with administering alternative protocols.	Patient education, patient assessment, administration, and patient communication	A survey of nurse unit managers. Focus groups with chemotherapy nurses.	Nurse unit managers (NUMs) (*n* = 6) Registered nurses (*n* = 36)	Six chemotherapy centres	Four task types (patient education, assessment, administration and communication) were identified as being associated with administering chemotherapy. Each patient received 3.3 h of staff time (1.7 h of direct contact time and 1.6 h of noncontact time) Interpretation: The identified tasks and time allocation highlight the complexity of chemotherapy protocols.
Green et al. (2012) Canada	To develop regimen‐based resource intensity weights for engagement in outpatient chemotherapy.	A mixed methods study	Methodology for determining regimen workload	To determine the nursing care required to manage patients receiving complex chemotherapy regimens, including the elements of direct and indirect care.	13 items (A‐M) The final calculation to determine Nursing Intensity Time was realized by a formula.	The consensus‐based approach was chosen with an expert panel.	Expert (*n* = unknown)	Outpatient chemotherapy	The final nurse intensity time demonstrates that regimens require different times for patient care. Interpretation: The nursing intensity methodology underscores the variability in care demands across chemotherapy regimens.
de Souza et al. (2014) (Brazil)	To evaluate the workload and productivity of nurses working in an oncology outpatient clinic.	An observational study Data collection: use the revised measure to collect data for 5 days. Single centre	N/A	To identify the time spent by nurses on activities during the care process (direct care, indirect care, personal activities, and associated activities).	Inter‐observer agreement was calculated at 88.5%.	An instrument was created combining interview, document analysis and a questionnaire. The adaptation: two meetings with the nurses. A pilot study was conducted.	Registered nurses (*n* = 7)	Oncology outpatient's clinic, 130 beds	Nurses performed more indirect care interventions/activities (40.2%) and spent more time on these activities (43.2%). Direct care activities accounted for 33.6%. Interpretation: It highlights the significant burden of indirect care activities in SACT nursing, emphasizing the need to address inefficiencies to optimize patient‐centred care.
Martin and Gaidzinski (2014) Brazil	To validate an instrument for measuring the time spent by nursing staff in the interventions/activities.	A cross‐sectional study Single centre	N/A	To identify what activities interfere with the workload of a nursing team in an outpatient unit.	An instrument with 26 items	An instrument consisting of 32 interventions was created, each activity was sought for a consensus on answers. Delphi method	Judges (*n* = 5)	Oncology and Hematology Outpatient Service	84% of the essential NIC interventions being validated at the workshop. The essential interventions for specialties provide reference for research. Interpretation: The validated instrument provides a structured approach to identifying workload interferences, enabling better management of nursing tasks in SACT settings.
Tuna et al. (2015) Turkey	To create a planning system for nursing staff size for an outpatient chemotherapy unit at a university hospital.	An observational study Data collection: 5 self‐developed tools were used for 5 weeks: 1) nurse profile, 2) chemotherapy practice, 3) venous port form, 4) blood products transfusion form, 5) intramuscular, subcutaneous implementation monitoring form. Single centre	Magnuson Model patient classification system (Cusack et al. 2004)	To determine the nursing staff size required according to workload as determined within the unit in line with patient classification	Six‐point acuity level (1–6)	N/A	Registered nurses (*n* = unknown)	An outpatient chemotherapy unit	Researchers found that dose calculations 67.1 s, medication preparation 200.47 s, cases of medication spill 106.71 s, informing the patient prior to treatment 282.67 s, establishing vascular access 150.13 s, management of side effects of treatment 950 s, post‐treatment patient education 150.58 s. Interpretation: Detailed time analysis for specific nursing tasks highlights critical areas for optimizing workflow and improving efficiency.
Vortherms et al. (2015) USA	To implement an evidence‐based oncology outpatient staffing system equity in workload distribution.	A cross‐sectional study Data collection: 1) The acuity‐based staffing tool was fully implemented for six‐week; 2) A short survey was distributed for feedback. Single centre	Acuity‐Based Tool	To create a point‐based system and identify examples of typical daily assignments.	Each patient is assigned an acuity by adding points (maximum 5 points per patient). An average assignment of 16 points per RN.	An evidence‐based practice (EBP) team to develop the tool. A six‐month pilot was completed comparing a control group to a pilot group.	Registered nurses (*n* = unknown)	Outpatient oncology chemotherapy infusion centre, 35 chairs	An 8% increase in patient activity with no additional RN staff needed to provide the care. Interpretation: The implementation of an acuity‐based staffing tool improved workload distribution, highlighting its potential for enhancing equity in SACT nursing assignments.
Baril et al. (2016) (Canada)	To test if better appointment planning and scheduling can evenly distribute the nurse workload without adding resources for the nurse's non‐value‐added tasks.	An observational study The time spent on patient was observed using work sampling technique, 4 days for the observation. Single centre	N/A	N/A	N/A	N/A	Patients (*n* = 159)	A hematology‐oncology clinic with 16 chairs	Time spends on value‐added tasks 27%, non‐value‐added tasks necessarily by the nurse 44%, non‐value‐added tasks not necessarily by the nurse 21%. A nurse is in a busy state for 92% of the time, inactive (unavailable) for 8% of the time. Interpretation: The study reveals that a significant portion of nurses' time in SACT units is spent on non‐value‐added tasks.
Edwards (2017) USA	To increase chair use and to maximize scheduled (patient) hours per day.	Pre‐Post study ABS was implemented with a rapid cycle test of change approach. Single centre	Acuity‐based scheduling (ABS) Informed by Vortherms et al. (2015) and West & Sherer (2009)	To assign patient acuity levels by length and type of treatment, equally distribute workload.	Six‐point acuity level (1–6) A target acuity level of no greater than 20 per day	ABS was adapted by nurses, administrators, and pharmacists	Registered nurses (*n* = unknown)	Cancer Centre infusion room	The entire ambulatory treatment centre is designated as main infusion (67%), level 1 (17%), and level 2 (16%). Interpretation: the adaptation process of the measure was not clear.
Santos and Gaidzinski (2019) Brazil	To apply the method developed by the WHO, called Workload Indicators of Staffing Need (WISN) for dimensioning the nursing staff for the care of cancer patients.	A quantitative, observational, documentary field study Single centre	Dimensioning of nurses of the chemotherapy outpatient clinic according to the WISN method	To reflect the work dynamics of the nursing staff.	35 nursing interventions and three activities (personal, standby and movement).	The instrument was validated during a four‐hour face‐to‐face workshop by judges.	Registered nurses (*n* = 17) Nursing technicians (*n* = 12)	Chemotherapy outpatient clinic	The working time was distributed as: nursing interventions of 88.5%, personal activity of 8.2%, standby time of 3.1% and movement of 0.1%. The WISN method proved to be applicable to the CT outpatient clinic. Interpretation: the study indicated that nurses were extremely busy with the current workload.
Cheevers et al. (2020) UK	To adapt the Registered Nurse Forecast (RN4CAST) nurse survey making it appropriate to assess the working environments of ambulatory chemotherapy nurses.	A mixed methods feasibility study Data collection: RN4CAST‐chemotherapy ambulatory tool (CAT) for 2 days Single centre	RN4CAST‐CAT	To assessed (1) the quality and safety of nursing care provided and (2) nursing care activities left undone.	Three sections of questions: Section A, nurses work environment; Section B, nurse's workload and patient safety, Section C, nursing activities left undone.	Literature review or as advised by expert opinion of two Advanced Nurse Practitioners. Cognitive interviews with nurses.	Registered nurses Cognitive interview (*n* = 6) Feasibility study (*n* = 12)	Ambulatory chemotherapy unit	The left undone care items with the highest rates: verbal communication with patients; checking how patients were managing any comorbidities. 54% of nurses not asking patients about specific toxicities before chemotherapy at least once a month or more. Interpretation: The high frequency of left undone care items, suggests workload pressures that compromise essential nursing tasks in SACT units.
Knox and Larmet (2021) Canada	To develop a Resource Intensity Weighting (RIW) tool to identify the time requirements for Ambulatory Care Unit (ACU) nursing activities.	A mixed methods study Single centre	Ambulatory Care Unit RIW Tool Adapted from Green et al. (2012)	To gather data on the time required to complete each of the RIW categories in the ambulatory care unit.	12 workload items Workload items for each patient are added to a formula to the time that patients required from nurses.	Nurse (*n* = 6) focus group identified the initial draft of the ACU RIW tool workload categories. A survey to gather data on the time required to complete each of the RIW categories in the ACU.	Registered nurses For survey (*n* = 14)	Ambulatory cancer care Unit	85% of the new patient group and 90% of the follow‐up group could be classified as low care needs. Interpretation: The RIW tool effectively stratifies workload, aiding workload distribution

## Appendix B Data Extraction Table for Qualitative Study

**Table:**

Author/Date country	Study aim	Methods	Setting/Sample	Themes identified	Nursing workload	Interpretation
Kamimura et al. (2012) USA	To examine the features of nursing practice environments that contribute to quality patient care and nursing job satisfaction.	Qualitative study with 2 focused group. A semi‐structured moderator guide was used. Thematic analysis	Ambulatory oncology setting Registered nurse (*n* = 13) across 9‐conuty. Eligibility criteria: current registered nurse license and employment in an ambulatory oncology setting as a direct care provider for 16 or more hours per week.	Practice Environments: Workloads, Support, and Resources Communication With Colleagues: Integral to Patient Care Negative Consequences of Unfavorable Nursing Practice Environment Features Positive Impacts of Practice Environments and Communication	Nurses' workloads are more challenging due to temporary shocks of variables such as vacation, resignation, and sick leave. Nurses think the increased workload may be due to uneven distribution. Poor quality of care and job dissatisfaction may result from high patient workload and uneven distribution of patients. The involvement of supportive staff can reduce the workload of nurses.	Workload challenges in ambulatory oncology settings are exacerbated by staffing shortages and uneven task distribution, indicating the need for better resource allocation to reduce nurse strain.
Bao et al. (2024) UK	To identify what systemic anti‐cancer therapy (SACT) day unit nurses' roles and responsibilities are so that they can be better recognized, resourced, and supported.	Qualitative study with semi‐structured interview A topic guide was used Thematic analysis.	SACT day units Registered nurse (*n* = 15) Eligibility criteria: UK registered nurses, currently employed in a UK SACT day unit and had worked in SACT day units for > 6 months.	SACT day unit nurses' roles: the specialist role, leadership role, enabling work role, and supportive role. Factors that affect SACT day unit nurses' roles: workforce shortages, work overload, treatment delays, and lack of recognition and support	Study identified the complexity of the workload for a single SACT day unit's patient. Nurses were forced to prioritize tasks with the heavy workload, resulted in deprioritised psychosocial support. Some patients come in for treatment without any pre‐SACT education which increases their workload on the day The number of patients being treated in SACT day units has increased considerably.	Nurses face complex workloads due to workforce shortages, treatment delays, and inadequate pre‐treatment patient education, emphasizing the need for better workload management and support systems to ensure quality patient care.
