# Supplementary material for: Nursing Workload in Systemic Anti‐Cancer Therapy Day Units: A Scoping Review and Gap Analysis
Source: Nurs Health Sci. 2026 Apr 19;28:e70330. doi: 10.1111/nhs.70330 (PMC13092646; doi:10.1111/nhs.70330)
Supplement: Supplementary file 2 — Data S2: Search terms for electronic databases. [file NHS-28-e70330-s003.docx]

Supplementary 2. Search terms for electronic databases

| CINAHL® | |
| --- | --- |
| Search | Query |
| #1 | (MH "Patient Acuity") OR (MH "Workload") OR (MH “stress”) OR (MH "Resource Allocation") |
| #2 | TI ( “Patient-acuity*” OR “patient* classif*” OR acuity* OR “safe staffing*” OR “trend* care*” OR caseload* OR “effective staffing*” OR “work load*” OR workload* OR demand* OR pressure OR Stress OR activit* OR Task* OR “case load*” OR “patient* severity” OR “patient* intensity” OR “treatment* complexit*” OR “patient* volume*” OR “patient* hours per day*” OR “patient safety*” OR “patient* depend*” OR activit*)  OR  AB (“Patient-acuity*” OR “patient* classif*” OR acuity* OR “safe staffing*” OR “trend* care*” OR caseload* OR “effective staffing*” OR “work load*” OR workload* OR demand* OR pressure OR Stress OR activit* OR Task* OR “case load*” OR “patient* severity” OR “patient* intensity” OR “treatment* complexit*” OR “patient* volume*” OR “patient* hours per day*” OR “patient safety*” OR “patient* depend*”) |
| #3 | #1 OR #2 |
| #4 | (MH "Oncology Service, Hospital") OR (MH "Integrative Oncology") OR (MH "Ambulatory Care Facilities") OR (MH "Ambulatory Care") OR ((MH "Outpatient Clinics, Hospital") AND (MH "Neoplasms")) |
| #5 | TI ((day N2 (oncol* OR infusion* OR surg* OR service*  OR unit* OR ward* OR support* OR transfusion* OR clinic* OR therap* OR treat* OR hospital* OR care OR nursing OR infusion)) N2 (oncolog* OR cancer* OR malignan* OR tumour* OR tumor* OR neoplasm* OR carcinogen* OR chemo* OR antineoplastic* OR “anticancer*” OR “precancer*” OR carcinoma* OR metastas* OR “neurotoxic drug*” OR cytotoxic OR immunotherap* OR “targeted therap*” OR apheresis OR plasmapheresis OR lymphoma* OR ocancer* OR sarcoma*) )  OR  AB ((day N2 (oncol* OR infusion* OR surg* OR service*  OR unit* OR ward* OR support* OR transfusion* OR clinic* OR therap* OR treat* OR hospital* OR care OR nursing OR infusion)) N2 (oncolog* OR cancer* OR malignan* OR tumour* OR tumor* OR neoplasm* OR carcinogen* OR chemo* OR antineoplastic* OR “anticancer*” OR “precancer*” OR carcinoma* OR metastas* OR “neurotoxic drug*” OR cytotoxic OR immunotherap* OR “targeted therap*” OR apheresis OR plasmapheresis OR lymphoma* OR ocancer* OR sarcoma*) ) |
| #6 | TI ((outpatient* OR ambulat* OR outreach* OR “home based” OR “day-base*” OR “out patient*” OR “systemic therapy ambulator*” OR  “systematic anti-cancer unit*” OR “infusion centre*” OR “infusion clinic*” OR “sact suite*” OR “infusion suite*” OR prehabilitat* OR sact OR sacts OR “stem cell harvest*” OR transplant* OR day unit* OR day care* OR day center*) N2 (oncolog* OR cancer* OR malignan* OR tumour* OR tumor* OR neoplasm* OR carcinogen* OR chemo* OR antineoplastic* OR “anticancer*” OR “precancer*” OR carcinoma* OR metastas* OR myeloma* OR “neurotoxic drug*” OR cytotoxic OR immunotherap* OR “targeted therap*” OR apheresis OR plasmapheresis OR lymphoma* OR ocancer* OR sarcoma*) OR sact OR “systematic anti-cancer*” OR ”comprehensive Treatment*”)  OR  AB ((outpatient* OR ambulat* OR outreach* OR “home based” OR “day-base*” OR “out patient*” OR “systemic therapy ambulator*” OR  “systematic anti-cancer unit*” OR “infusion centre*” OR “infusion clinic*” OR “sact suite*” OR “infusion suite*” OR prehabilitat* OR sact OR sacts OR “stem cell harvest*” OR transplant* OR day unit* OR day care* OR day center*) N2 (oncolog* OR cancer* OR malignan* OR tumour* OR tumor* OR neoplasm* OR carcinogen* OR chemo* OR antineoplastic* OR “anticancer*” OR “precancer*” OR carcinoma* OR metastas* OR myeloma* OR “neurotoxic drug*” OR cytotoxic OR immunotherap* OR “targeted therap*” OR apheresis OR plasmapheresis OR lymphoma* OR ocancer* OR sarcoma*) OR sact OR “systematic anti-cancer*” OR ”comprehensive Treatment*”) |
| #7 | #4 OR #5 OR #6 |
| #8 | #3 AND #7 |
