# Supplementary material for: Nursing Workload in Systemic Anti‐Cancer Therapy Day Units: A Scoping Review and Gap Analysis
Source: Nurs Health Sci. 2026 Apr 19;28:e70330. doi: 10.1111/nhs.70330 (PMC13092646; doi:10.1111/nhs.70330)
Supplement: Supplementary file 3 — Data S3: Simplified search term. [file NHS-28-e70330-s002.docx]

Supplementary 3 Simplified search terms

| #1 | Nursing |
| --- | --- |
| #2 | Acuity tool, Workload, Resource Allocation |
| #3 | Ambulatory Care, systematic anti-cancer unit, Oncology outpatient, outpatient, infusion room, clinic, cancer centre, Oncology Service |
